# The Combination of Sorafenib and PGV-1 Inhibits the Proliferation of Hepatocellular Carcinoma Through c-Myc Suppression in an Additive Manner: In Vitro Studies

**DOI:** 10.1155/adpp/4297953

**Published:** 2024-11-26

**Authors:** Dhania Novitasari, Ikuko Nakamae, Noriko Yoneda-Kato, Jun-ya Kato, Yoshitaka Hippo, Yusuke Suenaga, Dyaningtyas Dewi Pamungkas Putri, Edy Meiyanto, Muthi' Ikawati

**Affiliations:** ^1^Laboratory of Tumor Cell Biology, Division of Biological Science, Graduate School of Science and Technology, Nara Institute of Science and Technology, Nara, Japan; ^2^Cancer Chemoprevention Research Center, Faculty of Pharmacy, Universitas Gadjah Mada, Yogyakarta, Indonesia; ^3^Department of Pharmaceutical Analysis and Medicinal Chemistry, Faculty of Pharmacy, Universitas Padjadjaran, Sumedang, Indonesia; ^4^Department of Molecular Carcinogenesis, Chiba Cancer Centre Research Institute, Chiba, Japan; ^5^Laboratory of Evolutionary Oncology, Chiba Cancer Centre Research Institute, Chiba, Japan; ^6^Department of Pharmacology, Toxicology, and Clinical Pharmacy, Faculty of Pharmacy, Universitas Gadjah Mada, Yogyakarta, Indonesia; ^7^Department of Pharmaceutical Chemistry, Faculty of Pharmacy, Universitas Gadjah Mada, Yogyakarta, Indonesia

**Keywords:** combination therapy, curcumin analog, liver cancer, sorafenib

## Abstract

Hepatocellular carcinoma (HCC) is one of the most aggressive types of liver cancer, and it is frequently associated with upregulated c-Myc expression. Sorafenib (Sor) is commonly used to treat HCC, but many patients experienced mild to severe side effects due to prolonged Sor treatment during therapy. It has been known that Pentagamavunone-1 (PGV-1) exhibits a remarkable antiproliferative effect on several cancer cells, yet limited studies have reported its cellular activities in HCC. The current study aims to evaluate the anticancer effects of Sor in combination with PGV-1 on the progression of HCC proliferation. c-Myc expressing cells, JHH-7 and Huh-7, were used for this study, then Sor and PGV-1 were tested for their effect on the cellular physiology phenomena including cytotoxicity combination assay and colony formation assay, cell cycle profile and reactive oxygen species (ROS) level by flow cytometry, senescence induction by beta-galactosidase (SA-*β*-gal) assay, and migration inhibition by wound healing assay. The c-Myc expression was evaluated through Western blot. PGV-1 was more effective than Sor at inhibiting cell growth, and it showed greater selectivity for HCC over fibroblast cells. The combination of Sor with PGV-1 exhibited synergistic-additive cytotoxicity with an irreversible effect in HCC cell lines. The combination induced senescence similarly with Sor alone in JHH-7 cells, while PGV-1 enhanced the cellular senescence when combined with Sor in Huh-7 cells. Furthermore, the combination increased ROS level in the same way as PGV-1 did in HCC. The combination with PGV-1 acted better than Sor alone to inhibit JHH-7 cell migration. In addition, the combination treatment led to the suppression of c-Myc, particularly in JHH-7 cells. Taken together, combining Sor with PGV-1 promotes better efficacy than Sor alone to inhibit HCC cell proliferation, and further evaluation of the efficacy and safety of adding PGV-1 to Sor in HCC therapy is worthwhile as a potential combination treatment option.

## 1. Introduction

Hepatocellular carcinoma (HCC) is an aggressive form of liver cancer that often results in death [1]. In molecular level, HCC are characterized by their aggressiveness and the various and complicated mechanisms that contribute to it. Genetic analysis has shown that c-Myc amplification is detected in up to 70% of viral and alcohol-associated HCC [2]. In addition, the upregulated c-Myc predicts a more advanced and malignant phenotype, suggesting a pivotal role for c-Myc in the etiology of HCC [3, 4]. Furthermore, c-Myc is a desirable target because it not only regulates 20% of human genes but also controls cell cycle progression [5], senescence [6], cell migration [7], and the metabolic activity of cancer cells [8]. A recent discovery indicates that c-Myc expression levels also impact its cellular response, further complicating c-Myc-mediated carcinogenesis despite its short half-life (20–30 min) in nontransformed cells [9]. The presence of c-Myc amplification suggests the crucial function of c-Myc in the development of HCC, highlighting the urge to explore its potential as a novel therapeutic target [3]. In clinical practice, HCC is typically identified late, when surgical resection is no longer an option, despite being a very successful treatment for the initial stages of liver cancer [10].

Currently, small-molecule inhibitor sorafenib (Sor) is one of the gold standard treatments for HCC, as it suppresses the growth of cancer cells through multiple kinase pathways [11]. However, Sor is not without downsides and undesirable effects [12, 13], and it may be accountable for these unwanted consequences, necessitating the exploration of additional strategies to enhance the effectiveness of therapy without triggering additional side effects. Hence, the search continues for a small molecule with superior features and therapeutic efficacy against HCC that can be used for monotherapy or combination with Sor.

Pentagamavunone-1 (PGV-1) is a novel curcumin analog that has been proven as a promising anticancer drug candidate in several types of cancer [14, 15]. PGV-1 has shown greater anticancer efficacy than curcumin according to cancer cells and animal-based experiments [15, 16]. Cell cycle arrest at prometaphase is an essential feature of unique mechanism from PGV-1, following other cellular processes including induction of senescence and inhibition of cancer cell migration [14]. PGV-1 has been found to cause permanent mitotic arrest in hepatoma HepG2 and JHH-7 liver cancer cells [15, 16]. Furthermore, PGV-1 has been applied as co-treatment with chemotherapeutic drugs such as doxorubicin or 5-fluorouracil to increase cytotoxicity against breast [17] and colorectal cancer cells [18]. Here, we aim to examine the molecular mechanisms behind the anticancer effects of Sor and PGV-1 in human HCC. Based on the results of the current investigation, we propose that PGV-1 combined with the Sor may hold promise for the treatment of liver cancer.

## 2. Materials and Methods

### 2.1. Materials

Sor (purity 98%; SML2653) was acquired from Sigma (Missouri, USA). The synthesis and characterization of PGV-1 (purity 95%) was conducted at the Cancer Chemoprevention Research Center (CCRC) of Universitas Gadjah Mada (Yogyakarta, Indonesia) according to the protocol by Utomo et al. [19].

The human HCC cell lines JHH-7 (JCRB1031) and Huh-7 (JCRB0403) were acquired from the Japanese Cancer Resources Bank (JCRB) (Osaka, Japan). Dulbecco's modified Eagle's medium (DMEM) (#043-30085; Wako, Japan) was utilized for the cultivation of all cell types, as mentioned in a prior study [16]. The human dermal fibroblast (HDF) cells (C-12302) were procured from PromoCell (Heidelberg, Germany) together with the media provided by the manufacturer.

### 2.2. Methods

#### 2.2.1. Cell Viability Assay

The cells (2 × 10^3^ cells/well) were added to a 96-well plate and allowed to proliferate until they adhered to the surface. The stock solution of Sor or PGV-1 was dissolved in dimethyl sulfoxide (#043-07216; Wako, Japan), then diluted in a suitable culture medium, and subjected to a 72-h incubation period. Cell viability after treatment was evaluated using the Cell Counting Kit-8 (CCK-8; JE603) from Dojindo (Kumamoto, Japan), following the guidelines provided by the manufacturer. Subsequently, the absorbance values obtained from the microplate reader were converted into a percentage representing cell viability. These values were then used to calculate the half-growth inhibitory (GI_50_) score [15]. The quantification of the selectivity index (SI) involved calculating the GI_50_ value in the noncancer cell line (HDF) and dividing it by the GI_50_ value in the cancer cell lines (JHH-7 or Huh-7).

The combined effects of two compounds (PGV-1 and Sor) were assessed using a similar technique [17], later to be calculated for the combination index (CI) value based on the Chou–Talalay formula [20].

(1) $$\begin{array}{c} \text{CI}=\frac{D1}{D1x}+\frac{D2}{D2x}, \end{array}$$

where *D*1 and *D*2 are doses of compounds 1 and 2 combined to achieve *x*% inhibition, respectively, while *D*1*x* and *D*2*x* in the denominator reflected doses of compounds 1 and 2 to achieve *x*% (the particular percentage of inhibition when treated by a single compound) inhibition when treated alone. CI < 1, C = 1, and CI > 1 are defined as synergist, additive, and antagonist, respectively.

#### 2.2.2. Colony Formation Assay

A total of 5 × 10^3^ JHH-7 or Huh-7 cells were subjected to overnight incubation in 12-well plates, followed by treatment with 2.5 μM Sor and/or 0.25 μM PGV-1 for 72 h. Subsequently, the medium keeping the compound was disposed of and substituted with a fresh medium, wherein it was permitted to cultivate for an additional duration of 10 days. The cells were treated with a 4% paraformaldehyde solution for fixation and afterward stained using a 0.5% crystal violet solution [21]. The plates were documented for colony observation qualitatively.

#### 2.2.3. Cell Cycle Profile Analysis

The cell cycle distribution was evaluated following a 72-h treatment with 2.5 μM Sor and/or 0.25 μM PGV-1 using propidium iodide (PI) labeling. After the treatment, the cells (2 × 10^5^) were collected and incubated in PI (#P4170, Sigma, USA) solution (50 μg/mL in deionized water) containing Triton X-100 (#168-11805, Wako, Japan) and RNase (#12091-021, Invitrogen, Germany). The labeled cells were further processed using a flow cytometer (FACS Calibur, BD Biosciences) and examined to determine the distribution of each cell cycle phase using Cell Quest [15].

#### 2.2.4. Reactive Oxygen Species (ROS) Level Analysis

Further, we used dichlorofluorescein diacetate or DCFDA (#D6883; Sigma, USA) to determine ROS accumulation based on the oxidation of the DCFDA by radical oxygen (O•) within cells. The confluent cells were detached, then the cell suspension was diluted with 10% FBS in phosphate-buffered saline (PBS). Subsequently, 20 *μ*M DCFDA was added into the cells, followed by an additional incubation period of 30 min. The cell suspensions (5 × 10^4^ cells) were treated with 2.5 μM Sor and/or 0.25 μM PGV-1, then incubated for 24 h. The fluorescence of oxidized DCF was assessed using a flow cytometer (FACS Calibur, BD Biosciences). The intensity was calculated as the fold change relative to the untreated group [15].

#### 2.2.5. Cellular Senescence Assay

Following a 24 h treatment period with Sor and/or PGV-1 in HCC cells (2 × 10^5^/well), the cells were subjected to fixation using a 4% paraformaldehyde solution for 10 min. After washing with 1× PBS, the cells were stained with a 0.2% X-gal (#021-07852, Wako, Japan) which acts as a substrate for the *β*-galactosidase as a biomarker for cellular senescence. Subsequently, the cells were kept in a non–CO_2_ incubator overnight. Afterward, the cells were captured for subsequent analysis. The cells exhibiting a green-blue coloration were identified as positive senescent cells and further quantified across three distinct fields of view using ImageJ software [22].

#### 2.2.6. Wound Healing (Migration) Assay

Briefly, 5 × 10^4^ cells were introduced into each compartment of the insert cell culture system (#81176; Ibidi, Germany) and subjected to incubation for approximately 24 h. The medium was substituted with a deprived medium (0.5% of fetal bovine serum, FBS [#SH30070.03; Hyclone, USA]) and thereafter incubated overnight. On the following day, 10 *μ*g/mL Mitomycin C (#133-15931; Wako, Japan) that was diluted in the deprived medium was replaced with the insert culture. The solution was then stored for 2 h before being exposed to the compound (2.5 *μ*M Sor and/or 0.25 *μ*M PGV-1). Immediately following the treatment, the cells were observed and documented using a microscope, denoted as the initial time point (0 h). The cell migration was documented at certain time intervals (24, 42, and 48 h) to measure the rate of gap closing. This analysis was conducted using ImageJ software [14].

#### 2.2.7. Western Blot

JHH-7 and Huh-7 whole cells were collected after 72-h treatment with 2.5 *μ*M Sor and/or 0.25 *μ*M PGV-1, then the protein was lysed with lysis buffer. The equal amounts of protein were loaded for separation through 10%–15% SDS-PAGE gel, then the protein was transferred into PVDF membrane. Later, the membrane was incubated overnight by primary antibody, c-Myc (Abcam, #D84C12), and *γ*-tubulin (Sigma, #T6557) that served as housekeeping protein; the dilution was done as recommended by the manual sheet. The next day, the membrane was washed with PBS-Tween before probed with anti-rabbit (#NA9120V, Cytiva, UK) or anti-mouse (#NA931V, Cytiva, UK) secondary antibody for 1 h. The target protein was detected using ECL solution (#RPN2106, Cytiva, UK) for further exposure in an X-ray film (47410 26617, Fujifilm, Japan). The semiquantification for the protein band was employed using ImageJ.

#### 2.2.8. Statistical Analysis

The data from each group was expressed as mean ± standard deviation (SD). Comparisons among various groups were conducted using a one-way analysis of variance (ANOVA) followed by Tukey's post hoc test using GraphPad Prism (version 9.0). Statistical significance was determined by considering results with a *p*-value less than 0.05; the significance mark was indicated in compact letter display (CLD) within the graph.

## 3. Results

### 3.1. The Cytotoxic Effect of Sor and PGV-1 in Human HCC Cell Lines

**Figure 1:**
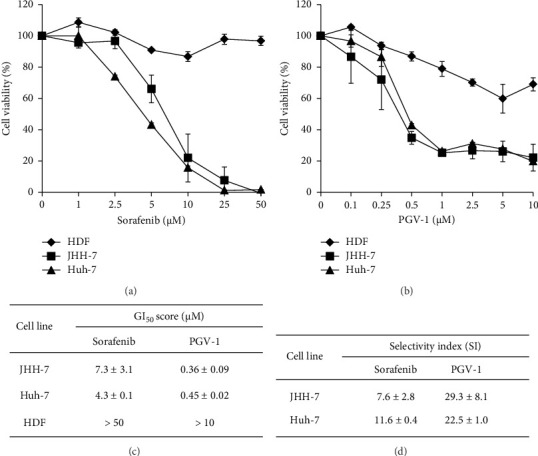
The antiproliferative effect of sorafenib and PGV-1 in hepatocellular carcinoma (HCC). (a) The percentage of cell viability from sorafenib and (b) PGV-1. (c) Half-inhibitory growth (GI_50_) scores of each compound in each cell line (*n* = 3). (d) Selectivity index (SI) of each compound by comparing the GI_50_ scores of normal cell line HDF and HCC cell line. The results in the graph were expressed as the mean ± SD (*n* = 3).

First, Sor and PGV-1 were tested individually to determine the effect on the growth of human HCC. Following a 72-h incubation period, it was determined that Sor exhibited sensitivity in both liver cancer cell lines (JHH-7 and Huh-7), while demonstrating lower levels of cytotoxicity in fibroblast cells. This conclusion was drawn based on the observation that the highest dose of Sor tested resulted in a slight inhibition of cell proliferation, as depicted in Figure 1(a). Remarkably, PGV-1 had a remarkable inhibitory effect exceeding 50% at a dose as low as 0.5 *μ*M. The viability of PGV-1 was relatively higher in fibroblast cells compared to its effects on cancer cells (Figure 1(b)). Moreover, the study demonstrated that PGV-1 exhibited significant cytotoxicity in both liver cancer cell lines, as shown by a GI_50_ score of less than 1 *μ*M. Notably, PGV-1 was over 20 times more effective than Sor in JHH-7 cells (Figure 1(c)). According to the SI calculation, PGV-1 exhibited an SI value of 29 in JHH-7 cells and 22 in Huh-7 cells, indicating that the PGV-1 demonstrated a high level of selectivity, particularly in HCC cells (Figure 1(d)).

### 3.2. The Cytotoxic Combination Effect of Sor and PGV-1 in Human HCC Cell Lines

**Figure 2:**
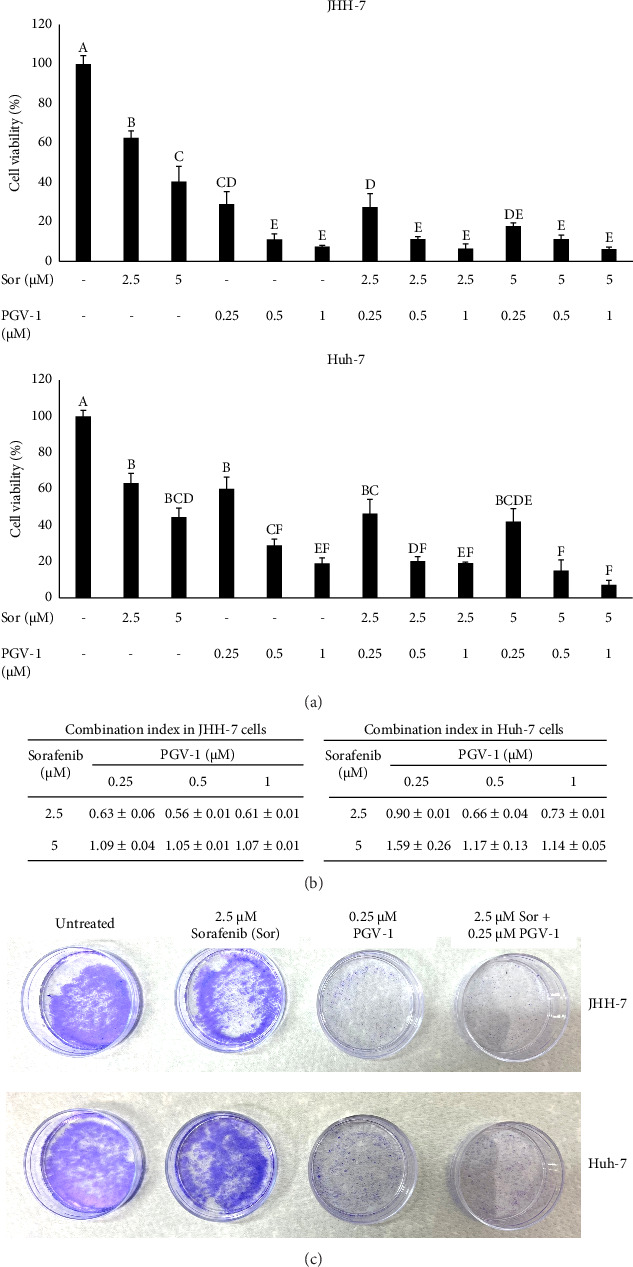
The combination treatment of sorafenib (Sor) and PGV-1 in JHH-7 and Huh-7 cells inhibited the proliferation irreversibly. (a) The percentage of cell upon combination in JHH-7 and Huh-7 cells. The results in the graph were expressed as mean ± SD (*n* = 3). Means denoted by a different letter indicate significant differences between treatments (*p* < 0.05). (b) The combination index (CI) of Sor and PGV-1 in JHH-7 and Huh-7 cells. (c) The representative qualitative colony formation assay in JHH-7 (upper) or Huh-7 (lower) cells after staining with crystal violet.

Following the single cytotoxic assay of Sor and PGV-1, we opted for two concentrations of Sor (2.5 and 5 *μ*M) and three for PGV-1 (0.25, 0.5, and 1 *μ*M) for determining the combination effect in JHH-7 and Huh-7 cells. While Sor demonstrated moderate cytotoxicity (percentage of viable cells 40%–50%), a single treatment of 0.25 *μ*M PGV-1 inhibited viable cell growth by 65% in JHH-7 cells. The combination of these compounds remarkably decreased the viable cells compared to the single treatment of Sor (Figure 2(a), upper graph). A similar effect was also reflected in the treatment of Huh-7 cells. Though it seemed that PGV-1 was less sensitive in Huh-7 cells, the combination treatment lowered its cell viability (Figure 2(a), lower graph). Based on the CI calculation, the combination with 2.5 *μ*M Sor resulted in a synergistic effect (CI < 1) in both cells, while the higher dose of Sor presented an antagonist effect (Figure 2(b)). Thus, we used 2.5 *μ*M Sor and 0.25 *μ*M PGV-1 in further evaluation. We treated with that combination for 3 days and replaced it with medium only (without the compound) throughout the rest of the 10 days; the combination treatment drastically inhibited colony formation of JHH-7 and Huh-7 cells compared with Sor-treated cells (Figure 2(c)). Based on these findings, the combination of Sor and PGV-1 caused irreversible growth in human HCC.

### 3.3. The Effect of Sor and PGV-1 Combination on Human HCC Cell Cycle Progression

**Figure 3:**
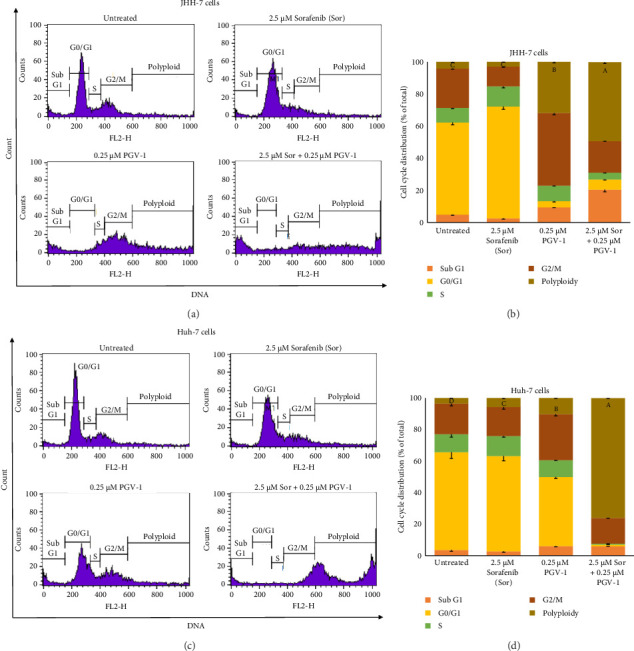
The cell cycle profile upon sorafenib (Sor) and PGV-1 treatment in HCC. (a) The flow cytogram of JHH-7 cells under 2.5 μM Sor, 0.25 μM PGV-1, and its combination treatment for 72 h. (b) The JHH-7 cells distribution after analysis in a flow cytometer (*n* = 3). (c) The same approach was also carried out for Huh-7 cells under similar conditions and determined by flow cytometry. (d) The Huh-7 cell distribution was visualized based on flow cytogram results (*n* = 3). The statistical analysis was conducted for polyploid phase toward all groups. Means denoted by a different letter in B and D indicate significant differences between treatments (*p* < 0.05).

The irreversible effect of the combination of Sor and PGV-1 on liver cancer cell proliferation prompted us to investigate its impact on cell cycle progression. The cell cycle profile was examined using a flow cytometer following a 3-day incubation period with the compound. It was observed that treatment with Sor resulted in an accumulation of cells in the G1 phase. Conversely, treatment with PGV-1 led to cell arrest in the G2/M phase and promoted polyploid accumulation in JHH-7 cells (Figures 3(a) and 3(b)). The combination treatment showed an increased accumulation of cells in both the mitotic and subG1 phases. At a dose of 2.5 *μ*M, Sor did not alter cell cycle progression in Huh-7 cells. However, even at a lower concentration of 0.25 *μ*M, PGV-1 remained sufficiently effective in inducing G2/M arrest. The combination significantly induced polyploid Huh-7 cell population, indicating cells arrested in mitosis (*n* > 2) (Figures 3(c) and 3(d)). The findings of this study suggest that PGV-1 plays a significant role in inducing cell cycle arrest when combined with Sor in human HCC.

### 3.4. The Effect of Sor and PGV-1 Combination on the Cellular Senescence and Intracellular ROS Level in Human HCC Cell Lines

**Figure 4:**
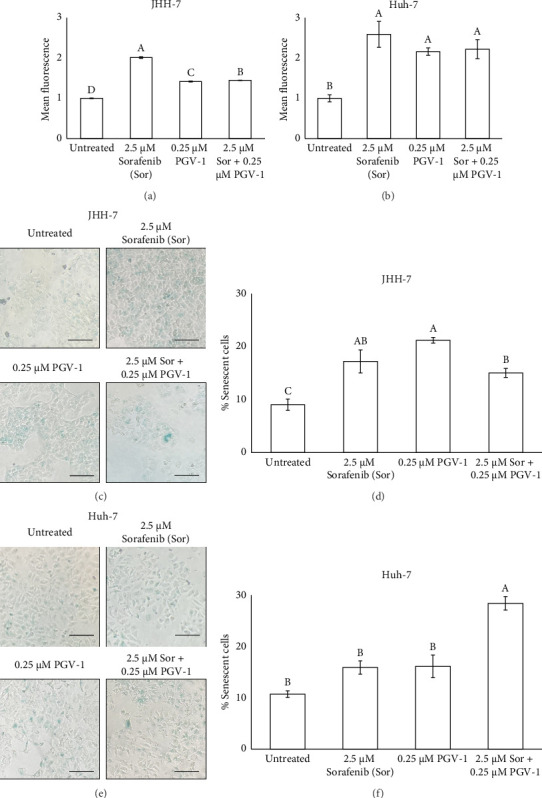
The effect of sorafenib (Sor) and PGV-1 combination upon intracellular ROS level and cellular senescence in HCC. (a) The quantification of fluorescent from DCF on JHH-7 and (b) Huh-7 cells. (c) The JHH-7 cells morphology after staining with 0.2% X-gal solution overnight. (d) The percentage of senescent JHH-7 cells. (e) A similar experiment was also conducted for treatment in Huh-7 cells, and the senescent cells were seen as the blue staining (scale bar = 100 μm). (f) The quantification of Huh-7 senescent cells per total cells. The results in the graph were expressed as the mean ± SD (*n* = 3). Means denoted by a different letter in graph A, B, D, and F indicate significant differences between treatments (*p* < 0.05).

Most chemotherapy treatments elevate ROS levels within cells, leading to a disturbance in the redox equilibrium in cancer cells due to the production of oxidative stress and ROS-induced cell damage. One of the consequences of the oxidative cellular is triggering cellular senescence, which has recently become the interesting target to eliminate cancer cells [23]. Thus, we questioned whether Sor and/or PGV-1 are also capable of inducing cellular ROS. The single Sor or PGV-1 treatment, as well as the Sor-PGV-1 combination, significantly increased cellular ROS compared to untreated JHH-7 (Figure 4(a)) and Huh-7 cells (Figure 4(b)), with Sor producing the highest cellular ROS. Collectively, this finding suggested that Sor and/or PGV-1 treatment induced intracellular ROS in HCC cells.

### 3.5. The Effect of Sor and PGV-1 Combination on Cellular Senescence in Human HCC Cell Lines

Since the combination of PGV-1 and Sor induced intracellular ROS in HCC, we further evaluated whether these treatments trigger the cellular senescence as one of the implications of higher ROS level. The results demonstrated that both single PGV-1 or Sor and its combination increased the senescent JHH-7 cells (Figures 4(c) and 4(d)). Still, the combination treatment did not result in a statistically significant increase in senescent cells compared to treatment with Sor alone.

In contrast, for Huh-7 cells, the combination treatment led to a more pronounced increase in cellular senescence than treatment with Sor alone, indicating that the combination therapy had a more significant effect on inducing senescence in this cell line (Figures 4(e) and 4(f)). These findings suggest that the impact of the treatments on cellular senescence may be cell line–specific, with combination therapy enhancing the senescence response in Huh-7 cells but not in JHH-7 cells.

### 3.6. The Effect of Sor and PGV-1 Combination on HCC Cell Migration

**Figure 5:**
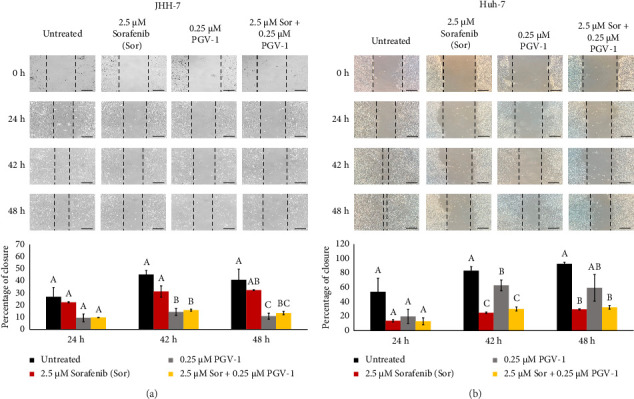
The antimigratory activity from sorafenib (Sor), PGV-1, and the combination upon migration in HCC. (a) Cells were documented for the migration during 24, 42, and 48 h after treatment on JHH-7 and (b) Huh-7 cells. The wound-free area was semiquantified with the ImageJ and converted into the closure percentage over the 0-h image (scale bar = 100 μm). The results in the graph were expressed as the mean ± SD (*n* = 3). Means denoted by a different letter in the graph indicate significant differences between treatments (*p* < 0.05).

Following the irreversible antiproliferative effect of Sor and PGV-1, we decided to observe whether the combination could also inhibit the cell migration in metastatic HCC. A single treatment with Sor did not much delay JHH-7 cell migration, while PGV-1 was more effective in inhibiting the migration. Further, the combination significantly inhibited the migratory activity in JHH-7 cells over 48 h of observation compared to a single Sor (Figure 5(a)). On the contrary, a different inhibitory effect of Sor and PGV-1 was demonstrated in Huh-7 cells, as seen by similar activity in the combination group with single Sor. Furthermore, Sor exhibited a stronger antimigratory effect than PGV-1 in Huh-7 cells (Figure 5(b)). The aforementioned observations indicated that Sor with PGV-1 was additively suppressing JHH-7 and Huh-7 cell migration.

### 3.7. The Effect of Sor and PGV-1 Combination on c-Myc Expression

**Figure 6:**
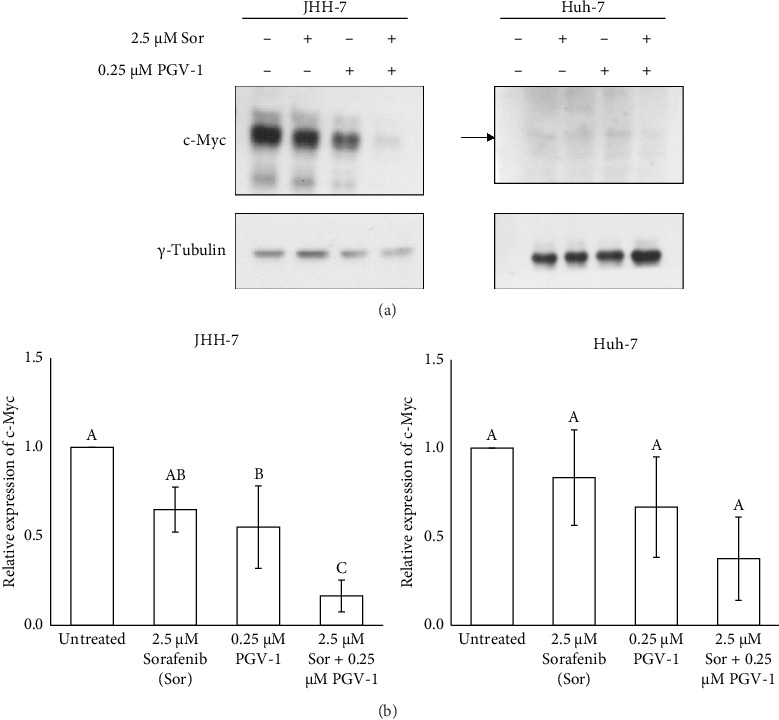
The effect of sorafenib (Sor)-PGV-1 combination in c-Myc protein level. (a) The expression level of c-Myc was detected by Western blotting after treatment for 72 h. (b) The analysis of relative expression of c-Myc protein after normalized with *γ*-tubulin (*n* = 3, independent experiments). Means denoted by a different letter in graph B indicate significant differences between treatments (*p* < 0.05).

In order to investigate the molecular mechanisms underlying the combined therapeutic effect of Sor and PGV-1, we assessed the expression levels of c-Myc protein in HCC cells treated with the drugs using Western blot (Supporting Figure 1). The combination of Sor-PGV-1 exhibited a significant reduction in c-Myc levels when compared to the single treatment of Sor in JHH-7 cells (Figure 6(a)). In contrast, the c-Myc protein exhibited no significant alterations following treatment in Huh-7 cells (Figure 6(b)). The findings of this study suggest that the downregulation of c-Myc expression may play a role in the molecular mechanisms behind the effects of Sor/PGV-1 in HCC.

## 4. Discussion

The present study reported that treatment with Sor suppressed the proliferation of HCC, and this effect was improved when combined with the mitotic inhibitor PGV-1. Sor has been chosen as a first-line small-molecule inhibitor for advanced-stage liver cancer due to its effect on interfering with multi-kinases [1]. But, many reports show that long-term therapy of Sor causes changes in kinase signaling, increased drug efflux, drug metabolism inside cells, etc. [11]. Thus, strategies to overcome the hurdles in treatment with Sor have been developed, including combining other chemotherapy with different targets or molecular activities in cancer cells. A recent study reported the second clinical trial phase of Sor with immune checkpoint inhibitor Toripalimab that demonstrated promising effectiveness and manageable side effects as a first treatment choice for patients with inoperable HCC, with some patients experiencing tumor downstaging and were able to undergo surgical removal of the tumor [24]. Still, several efforts need to be explored to find the potential combination therapy for HCC. The curcumin analog PGV-1 has been studied a lot in several types of cancer, and because it works so well against cancer, it has been studied in combination with existing chemotherapies [18, 25]. Furthermore, the latest studies have shown that a single treatment with PGV-1 inhibited liver cancer cells, notably in hepatoma HepG2 [15] and HCC JHH-7 cells [16]. Therefore, combining PGV-1 with Sor is expected to enhance the cytotoxic effect on HCC.

The current study showed that while Sor inhibited HCC populations by 50% in higher doses (around 5 *μ*M), PGV-1 exhibited more effectiveness even in concentrations less than 1 *μ*M. The result led us to investigate whether combining these drugs enhanced the efficacy of treating HCC. Indeed, 2.5 *μ*M Sor with 0.25–1 *μ*M PGV-1 exhibited a synergistic effect in both cell lines based on CI calculation, while the additive effect resulted in a higher dose of Sor (5 *μ*M). In addition, the combination with PGV-1 suppressed colony formation abilities in both cell lines. The potential reasons for the synergistic anticancer effects were then examined. The results demonstrate that Sor and PGV-1 alone prevented the cell cycle progression in the G1 and G2/M phases, subsequently. Interestingly, the combination treatment halted the cell cycle in the G2/M phase in HCC cells. Prior report revealed that Sor treatment suppressed the G1 phase protein cyclin D1 expression in HCC [26], while PGV-1 itself has been shown to promote mitotic arrest in liver cancer cells HepG2 and JHH-7 [15, 16]. This result suggested that cell cycle arrest was not the driving force behind the synergistic anticancer effects since the cells in G1 phase following the combination treatment almost diminished (Figure 3).

Our current results confirmed that both Sor and PGV-1 alone were able to induce senescence in JHH-7 and Huh-7 cells, but their synergistic efficacy in generating senescent cells was only found in Huh-7 cells. The ability of several cancer chemotherapies to elicit stress-induced senescence in cancerous cells is a relatively new but crucial strategy for improving treatment outcomes [27]. Moreover, selective removal of senescent cells, or “senolysis,” is gaining attention as an adjunct strategy. While senescence induction can suppress tumor growth, the persistence of senescent cells can promote a pro-tumorigenic environment over time due to chronic SASP (senescence-associated secretory phenotype) production, hence targeting and eliminating these cells after senescence induction may improve therapeutic outcomes by preventing recurrence and minimizing side effects. Study has shown that therapy-induced senescence can sensitize cancer cells to other treatments, making senescence-based strategies present a compelling opportunity for advancing anticancer therapies [28].

In regard to the effect on inducing ROS accumulation, it appears that PGV-1 may dominate the effect when combined with Sor in HCC, as the ROS intensity observed in the combination treatment was similar to that seen with PGV-1 alone. However, since we did not examine the molecular pathways involved in the increased ROS production, further investigation into these mechanisms would be valuable for understanding how the combination of PGV-1 and Sor induces ROS production in HCC cells. While several studies have shown a link between chemotherapy use and the tendency to generate ROS production [29] and the onset of premature senescence in cancer cells, our results indicate that cellular ROS production was not the only method by which senescence can be induced in HCC.

The combination also led to a decrease in cell migration in addition to its antiproliferative effects. This demonstrated that Sor alone was unable to impede JHH-7 cell migration but that adding PGV-1 to the mix significantly slowed the migration (just as PGV-1 alone did). In Huh-7 cells, however, we saw a quite distinct phenomenon: PGV-1 alone did not suppress cell migration nearly as much as Sor did, suggesting that their action was cumulative if compared to Sor itself. Sor is known to suppress matrix metalloproteinases (MMPs) expression in HepG2 cells [30], while PGV-1 is reported to reduce cell migration and invasion in hepatoblastoma HepG2 [15], suggesting that it may be useful to investigate their additive effect in malignant HCC.

Finally, our results showed that the combination inhibited c-Myc in JHH-7 cells while leaving it mostly undisturbed in Huh-7. Despite the fact that both cell lines have been shown to have a nonmutated c-Myc by transcriptomic analysis and share the same hepatoblast-like characteristics [31], they are classified as belonging to different subgroups by Boyault et al. [32]: JHH-7 is in G1 group (with a predominant phospho-ERK pathway) and Huh-7 belongs to G2 group (with Wnt and PI3K signaling as their main molecular features). Therefore, these factors may account for the varying cellular c-Myc protein levels that result from combination treatment. Indeed, c-Myc can be triggered in a variety of ways, leading to distinct biological functions such as cell proliferation (when activated via MAPK signaling) [33] and differentiation (when activated via the Wnt pathway) [34]. Studies have confirmed that c-Myc protein is expressed in Huh-7 cells, although the protein appears at a weaker intensity compared to JHH-7 cells. This suggests that the endogenous level of c-Myc in HCC may be influenced by the effects of PGV-1 and/or Sor in suppressing its expression. However, further evaluation using additional HCC cell lines is necessary to validate this hypothesis and determine whether this suppression is consistent across different models of HCC. Jindal et al. [35] reported that Sor was able to suppress c-Myc expression, and combination with multiple CDKs inhibitor milciclib showed a synergistic effect to reduce c-Myc protein in HCC. This time, the addition of mitotic inhibitor PGV-1 also synergistically suppressed c-Myc in JHH-7 cells. PGV-1 activity in mitosis may contribute to c-Myc degradation in human HCC, as c-Myc regulates cell cycle regulators and occurs at mitosis [5].

Various etiological variables and their underlying processes are typically responsible for HCC progression. It is fair to suggest that disrupting a particular pathway could trigger a compensatory signaling process, and the use of combination therapies involving drugs that have distinct mechanisms of action may result in improved effectiveness. This may be a plausible explanation for why patients with HCC acquire resistance to nearly all single chemotherapeutic drugs. In regard to our current result, it has been acknowledged that c-Myc promotes the aggressiveness of HCC [4], and including c-Myc degradation may be an effective strategy for eradicating these tumors. Notably, c-Myc inhibition increases the sensitivity of chemotherapeutic drugs to HCC [3]. Our results presented another promising approach to treat liver cancer that target c-Myc that hopefully can be beneficial to enhance the efficacy of Sor.

Since the current study did not observe how the combination affects the HCC growth in vivo, the optimum dosage form of each drug could not be justified. Still, prior result already demonstrated that PGV-1 suppressed growth in colon, leukemia, and breast tumors, and also prevented liver carcinogenesis in dimethylhydrazine-induced rats [36]. Hence, PGV-1 still offers potential to be used as combination treatment with Sor in HCC.

## 5. Conclusion

The current study highlights that combining Sor and PGV-1 exhibits synergistic anticancer effects on suppressing the proliferation in human HCC JHH-7 and Huh-7 cells, inhibits colony formation and cell migration, and also enhances intracellular ROS generation to partly mediate chemotherapy-induced senescence in liver cancer cells. Therefore, Sor and PGV-1 together might serve as a promising approach for treating patients with liver cancer.

## Data Availability

The datasets used to support the findings of this study are available from the corresponding authors upon request.

## Supporting Information

Supporting Figure 1: Western Blot for C-myc expression in HCC (JHH-7 and Huh-7 cells) following Sor and PGV-1 treatment.
