# Supplementary figures and images for: The Combination of Sorafenib and PGV-1 Inhibits the Proliferation of Hepatocellular Carcinoma Through c-Myc Suppression in an Additive Manner: In Vitro Studies
Source: Adv Pharmacol Pharm Sci. 2024 Nov 26;2024:4297953. doi: 10.1155/adpp/4297953 (PMC11614502; doi:10.1155/adpp/4297953)

Western Blot result

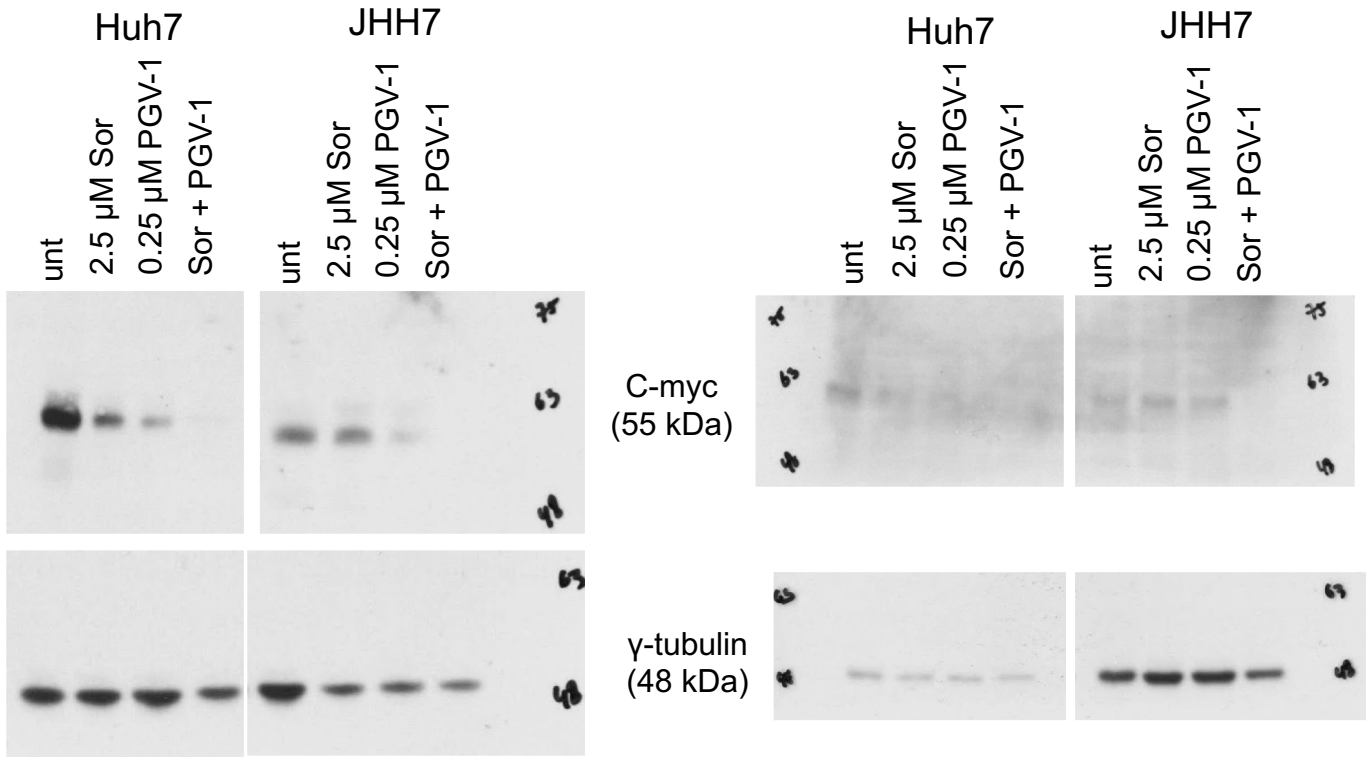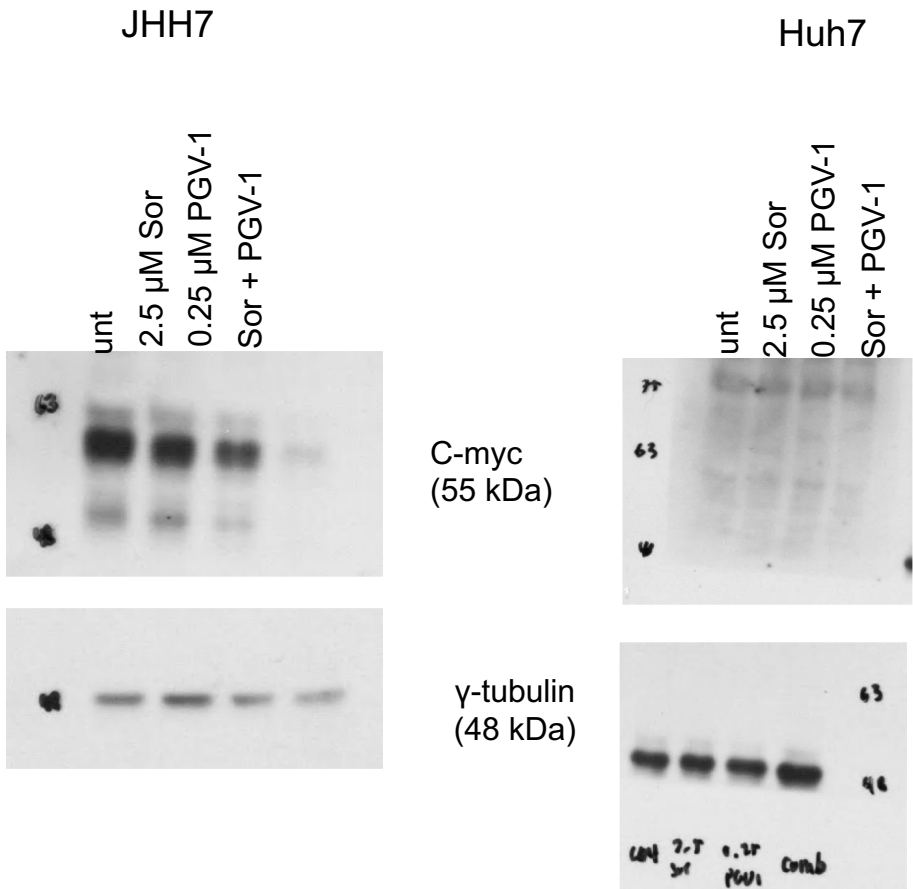

Supplement: Supporting Information — Additional supporting information can be found online in the Supporting Information section. [file 4297953.f1.pdf]
